# Genetic homogeneity of North-African goats

**DOI:** 10.1371/journal.pone.0202196

**Published:** 2018-08-16

**Authors:** Nadjet-Amina Ouchene-Khelifi, Mohamed Lafri, François Pompanon, Abdessamad Ouhrouch, Nassim Ouchene, Véronique Blanquet, Johannes A. Lenstra, Badr Benjelloun, Anne Da Silva

**Affiliations:** 1 Science Veterinary Institute, University of Blida, Blida, Algeria; 2 Laboratory of Biotechnology related to Animal Reproduction (LBRA), University of Blida, Blida, Algeria; 3 Univ. Grenoble-Alpes, Univ. Savoie Mont Blanc, CNRS, LECA, Grenoble, France; 4 National Institute of Agronomic Research (INRA Maroc), Regional Center of Agronomic Research, Beni Mellal, Morocco; 5 Laboratoire de Biotechnologies et Valorisation des Ressources Phytogénétiques (LBVRP), Université Sultan Moulay Slimane, Béni Mellal, Maroc; 6 Univ. Limoges, INRA, EA7500, USC1061 GAMAA, Limoges, France; 7 Utrecht University, Faculty of Veterinary Medicine, Utrecht, Netherlands; Fred Hutchinson Cancer Research Center, UNITED STATES

## Abstract

North Africa represents a rich and early reservoir of goat genetic diversity, from which the main African breeds have been derived. In this study, the genetic diversity of four indigenous Algerian goat breeds (*i*.*e*., Arabia, Makatia, M’Zabite and Kabyle, with n = 12 for each breed) has been investigated for the first time by genome-wide SNP genotyping; moreover in a broader context, genetic structuration of Algerian and Moroccan goats was explored (*via* F_ST_, MDS, STRUCTURE, FineSTRUCTURE, BAPS, sPCA and DAPC analyses). At national level, the study revealed high level of genetic diversity and a significant phenomenon of admixture affecting all the Algerian breeds. At broader scale, clear global genetic homogeneity appeared considering both Algerian and Moroccan stocks. Indeed, genetic structuration was almost nonexistent among Arabia (from Algeria), Draa, Black and Nord (from Morocco), while the ancestral Kabyle and M’Zabite breeds, reared by Berber peoples, showed genetic distinctness. The study highlighted the threat to the Maghrebin stock, probably induced by unsupervised cross-breeding practices which have intensified in recent centuries. Moreover, it underlined the necessity to deepen our understanding of the genetic resources represented by the resilient North-African goat stock.

## Introduction

About 11,000 years ago, Neolithic farmers in the Near East, started keeping herds of wild bezoar ibex (*Capra aegagrus*) for their milk, meat, hair and skin [1–4]. Thus began one of the oldest histories of domestication which led to the development of the domestic goat (*Capra hircus*). From the domestication centers in Central Zagros and Eastern Anatolia, goat rapidly spread westward along the Mediterranean coasts [5–7]. Archaeological remains, of both sheep and goat, found along the Mediterranean coast of North Africa, suggest a fast dispersal from Southwest Asia into North Africa between 7,000 BP and 6,000 BP [8]. According to bones from the Capeletti Cave, the presence of sheep and goat in Algeria dates back to 6,000 BP [8], and the Gueldaman Cave in the Kabylie Mountains reveals a well-established pastoral economy around 6400–5900 BP [9]. Subsequent waves of goat migrations from different genetic backgrounds might have influenced the Maghrebin livestock by leading to complex genetic make-up closely linked to the area’s history [10]. In particular, the Arabian invasion (around 1340 BP) is thought to have exerted a significant impact [11].

In Africa, southward movement of domestic goats only began 5,000 year ago, likely in response to climatic shifts ([7], [12–14]). Hence, Egypt, Sudan, Libya and the Maghreb (Algeria, Morocco, and Tunisia) constitute an original hotspot of goat genetic diversity from which main breeds of Africa have been derived. Using mtDNA and Whole Genome Sequencing (WGS) data, Benjelloun *et al*. [15] highlighted the remarkable genetic richness of indigenous Moroccan goat populations as well as their weak geographic structure. However, and in spite of their importance as genetic resource, the understanding of the Maghrebin goat diversity is still incomplete.

In this study, we assessed the genetic diversity of the four main indigenous Algerian goat breeds, using the Illumina SNP50K Genotyping BeadChip. These breeds are classified into three major types defined by Mason [16]: First, the Berber type, including the Kabyle breed, is probably the most ancestral type, showing skeleton traits of Neolithic goat, and is supposed to have arrived between 7,000 BP and 6,000 BP [8]. Second, the Sahelian type, including Arabia and Makatia breeds, has probably been introduced by a more recent east-west migration flow of goats with long legs and long pending ears. Makatia may have been derived from Arabia [17]. Third, the Nubian type, including the M’Zabite breed, with convex profile, is found mainly in Southern oases and probably originates from Egypt and Sudan [18]. We did not include in our study the several “exotic” breeds that are kept in the Maghreb area, including Saanen and Alpine (a few thousand heads), and a limited number of Murcia, Maltese, Toggenburg, Damasquine, Malagueña, and Andalouse breeds [19–20].

Our aims were (i) to provide the first comprehensive view of Algerian goat genetic diversity in comparison with that of Moroccan goat and (ii) to assess if the phenomenon of genetic dilution detected in the Algerian sheep [21–22], was also observed in the Maghrebin goat stock.

## Material and methods

### Ethics statement

The blood used for all of the analyses was collected by veterinarians during routine blood sampling, for medical care or follow-up, which did not require ethical authorization. All the samples and data processed in our study were obtained with the consent of the breeders.

### Breeds

The sample size was chosen in the light of the studies by Nazareno *et al*. [23] and Willing *et al*. [24]. For each of the four breeds (*i*.*e*. Arabia, Makatia, M’Zabite and Kabyle), we sampled 12 individuals, as much as possible from different flocks. If samples were obtained from the same farm, non-related individuals were selected on the basis of their pedigree (if documented) and/or information provided by the breeder (see S1 Table for GPS coordinates and breed descriptions).

Moroccan goats were characterized by WGS data produced by Benjelloun et al. [15] for the following breeds: Black (n = 22, distributed throughout Morocco), Draa (n = 14, mainly from the Southern Draa valley) and Nord (n = 8, mainly from the Rif area). Sampling details and GPS coordinates can be found in [15].

### Genotyping and SNP quality control

For the Algerian goats, blood samples were cryo-preserved until DNA extraction and analysis. Genomic DNA was purified from whole blood by protease K digestion and salting-out method [25]. All animals were genotyped for 53,347 SNPs, using the Illumina GoatSNP50K Genotyping BeadChip (Illumina, Inc.) and carried out by Van Haeringen Laboratorium (Wageningen, Netherlands). SNPs and animals were pruned using the following filtering parameters, in the PLINK v1.07 software [26]: (i) SNP call rate ≤97%, (ii) SNP minor allele frequency (MAF) ≤10%, (iii) animals with ≥10% missing genotypes, (iv) SNPs that did not pass the HWE test (P≥0.001).

For the Moroccan goats, 43,690 of the 53,347 BeadChip SNPs were derived from WGS data. A merged Moroccan and Algerian dataset retained 38,296 SNPs after quality control.

### Data analysis

#### Genetic diversity

The level of genetic diversity was assessed *via* the observed percentage of heterozygote genotypes per individual (H_o_) using PLINK.

#### Genetic structure

The extent of population subdivision was examined by calculating the global multi-locus F_ST_ values. The index of pair-wise F_ST_ of Weir and Cockerham [27] and their associated 95% confidence intervals were determined using the software Genetic Data Analysis (GDA) [28]. Multidimensional scaling (MDS) analysis was performed using PLINK. To ensure that uncorrected Linkage Disequilibrium (LD) did not distort the analysis, SNP pruning was used to identify a subset of SNPs using the—indep option of PLINK with the following parameters: 50 SNPs per window, a shift of five SNPs between windows, and a variation inflation factor’s threshold of two (corresponding to r^2^>0.5). Pair-wise Identical By State (IBS) distances were calculated for the pruned dataset. The graphical representation was depicted using the R (version 3.0.1 [29]) RColorBrewer package.

Genetic clusters of individuals were identified *via* a Bayesian model-based approach implemented in STRUCTURE 2.3.4 [30–33] using: admixture model, correlated allele frequencies, 50,000 burn-in followed by 100,000 simulations and K ranging from two to five. Convergence was checked using ten runs for each K value. The most probable value of K was estimated on the basis of the ΔK statistic [34]. The software CLUMPP ver. 1.1.1 [35] was used to align the repetitions for each K and the result was visualized by the program DISTRUCT ver. 1.1 [36].

For the approach of fineSTRUCTURE [37], based on haplotypes, the dataset was filtered and phased using SHAPEIT ver. 2 [38]. After removing seven individuals with *F*_IS_ >0.1 [39], we used CHROMOPAINTER [37] to analyze the painted data set in order to identify homogeneous clusters. Visualization of the posterior distribution of clusters was then performed using the tree-building algorithm of fineSTRUCTURE.

Patterns of migration were investigated using the Bayesian admixture analysis (with the predefined “populations” option) in BAPS 6.0 [40]. Parameter values were chosen following recommendations of Corander and Marttinen [41]. The function “Plot Gene Flow” was used to draw networks of clusters.

Spatial analysis of Principal Components (sPCA) was performed with the R package ADEGENET [42], using as connection network, the Delaunay triangulation [43]. Monte Carlo tests were used to check the statistical significance of the spatial structures (global and/or local spatial structure) for 10,000 iterations. The sPCA results were visualized by plotting the samples according to their geographic coordinates, with colors corresponding to their sPCA coordinates.

Discriminant Analysis of Principal Components (DAPC) was performed considering prior information on breeds, using the approach implemented in the ADEGENET package within the statistical R package. In addition, the optimal number of genetic clusters describing the data (*i*.*e*. without including prior information on breeds) was identified using the sequential K-means clustering algorithm. The different clustering solutions were compared using the Bayesian Information Criterion (BIC) and individuals were assigned to the inferred clusters.

## Results

### Genetic diversity

All 48 Algerian samples had a call rate ≥97% and were retained for further analyses. From the 53,347 SNPs 49,202 autosomal SNPs passed quality control. After LD-based correction suggested by López Herráez *et al*. [44], H_o_ calculated by breed, showed limited variability; average value was of 0.40 (s.d. = 0.02). The lowest number of SNPs not meeting quality check was observed for the Arabian goats (see S2 Table).

Moroccan breeds showed values of genetic diversity very close to those of Algerian breeds, with average observed heterozygosity (H_o_) ranging from 0.40 (Black and Draa) to 0.41 (Nord).

### Genetic structure

Considering all the Algerian breeds, the mean F_ST_ was 0.030 [0.028–0.031] (IC_95%_). Calculation of pair-wise F_ST_ (Table 1) was used to explore the genetic relationships among Algerian and Moroccan breeds. Considering Algerian breeds: M’Zabite was clearly distinct with F_ST_ values >0.40, whereas Arabia and Makatia were separated by the lowest pairwise F_ST_ value of 0.016. Considering breeds from both countries: The Algerian Arabia and the three Moroccan breeds, Draa, Black and Nord, showed extremely low values. The mean F_ST_, considering these four breeds was 0.005 [0.004–0.006] (IC_95%_).

**Table 1:** Pair-wise F_ST_ for Algerian and Moroccan goat breeds. In brackets 95% confidence intervals, in parentheses countries of origin.

	Arabia (Algeria)	Kabyle (Algeria)	M’Zabite (Algeria)	Draa (Morocco)	Northern (Morocco)
Kabyle (Algeria)	0.021 [0.018–0.022]				
M’Zabite (Algeria)	0.037 [0.034–0.038]	0.044 [0.041–0.046]			
Makatia (Algeria)	0.013 [0.011–0.014]	0.029 [0.026–0.030]	0.037 [0.034–0.038]		
Draa (Morocco)	0.008 [0.006–0.009]	0.025 [0.023–0.026]	0.045 [0.042–0.046]		
Northern (Morocco)	0.008 [0.006–0.009]	0.022 [0.019–0.023]	0.032 [0.029–0.033]	0.007 [0.005–0.009]	
Black (Morocco)	0.004 [0.003–0.004]	0.022 [0.020–0.023]	0.042 [0.040–0.043]	0.003 [0.002–0.004]	0.006 [0.004–0.007]

#### Relationships between Algerian breeds

**Fig 1:**
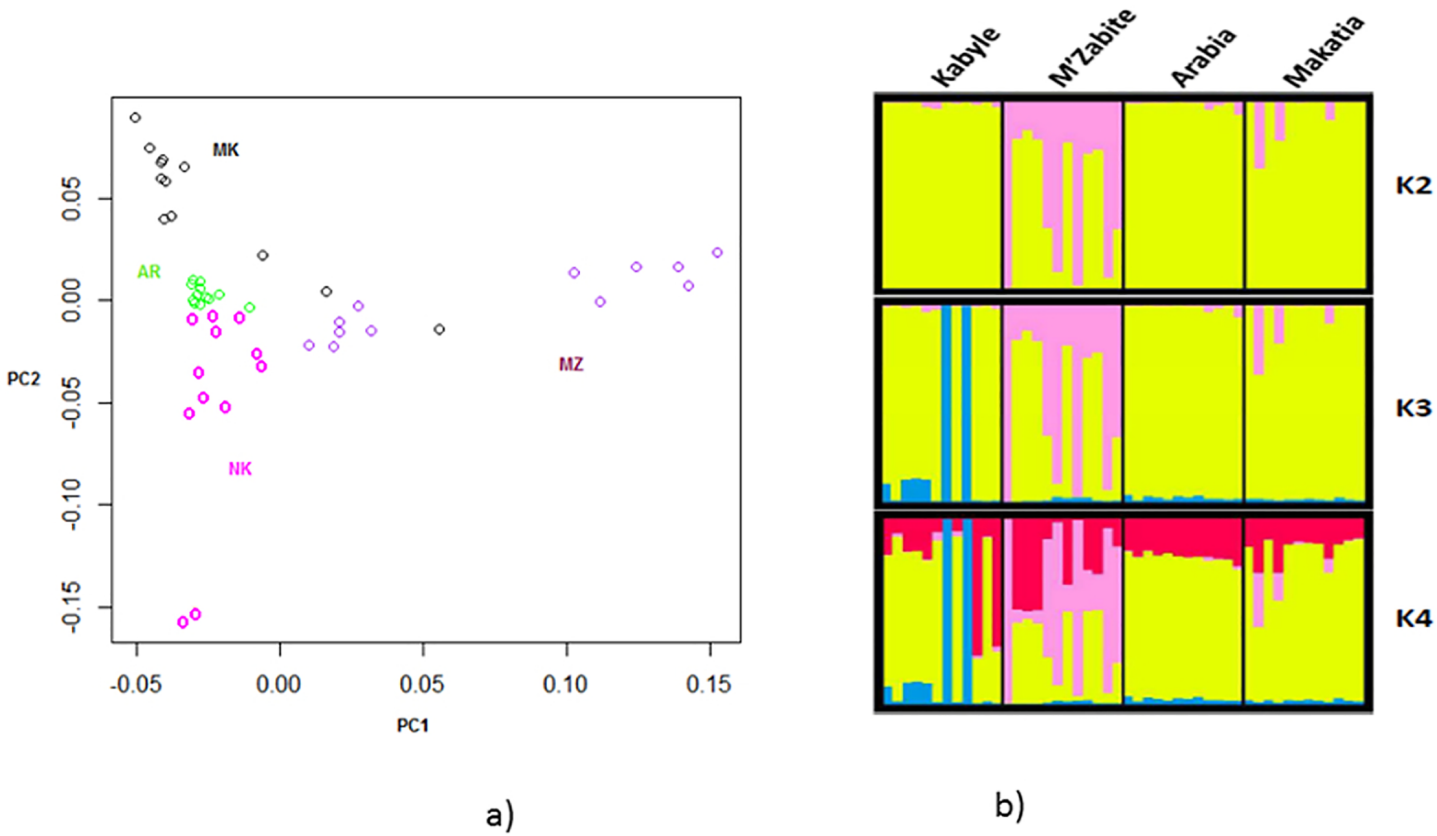
Genetic diversity of four local Algerian goat breeds; a) Multidimensional scaling analysis based on the matrix of pair-wise Identity By State (IBS) similarity scores; b) genetic structure inferred by Bayesian model-based clustering. K = number of clusters. The following abbreviations are used in the figure: MZ = M’Zabite; NK = Kabyle; AR = Arabia; MK = Makatia.

MDS analysis of the pair-wise IBS distances between Algerian goats (Fig 1A) showed a central position for Arabia with the three other breeds around it. Six M’Zabite and two Kabyle individuals appeared particularly distinct from the central core.

This pattern was confirmed by STRUCTURE analysis, assuming two to four clusters (Fig 1B). At K = 2, M’Zabite was partially differentiated from the others with the same six individuals, previously identified by MDS analysis, deviating from the others. At K = 3, the two Kabyle goats individualized by MDS analysis were separated (in blue) whereas other Kabyle goats still clustered with Arabia and Makatia (mainly in yellow). Even at K = 4, Arabia and Makatia remained indistinct from each other. The ΔK criterion [34] indicated K = 2 as the most likely subdivision, highlighting the genetic peculiarity of M’Zabite but also genetic overlap for the other breeds.

**Fig 2:**
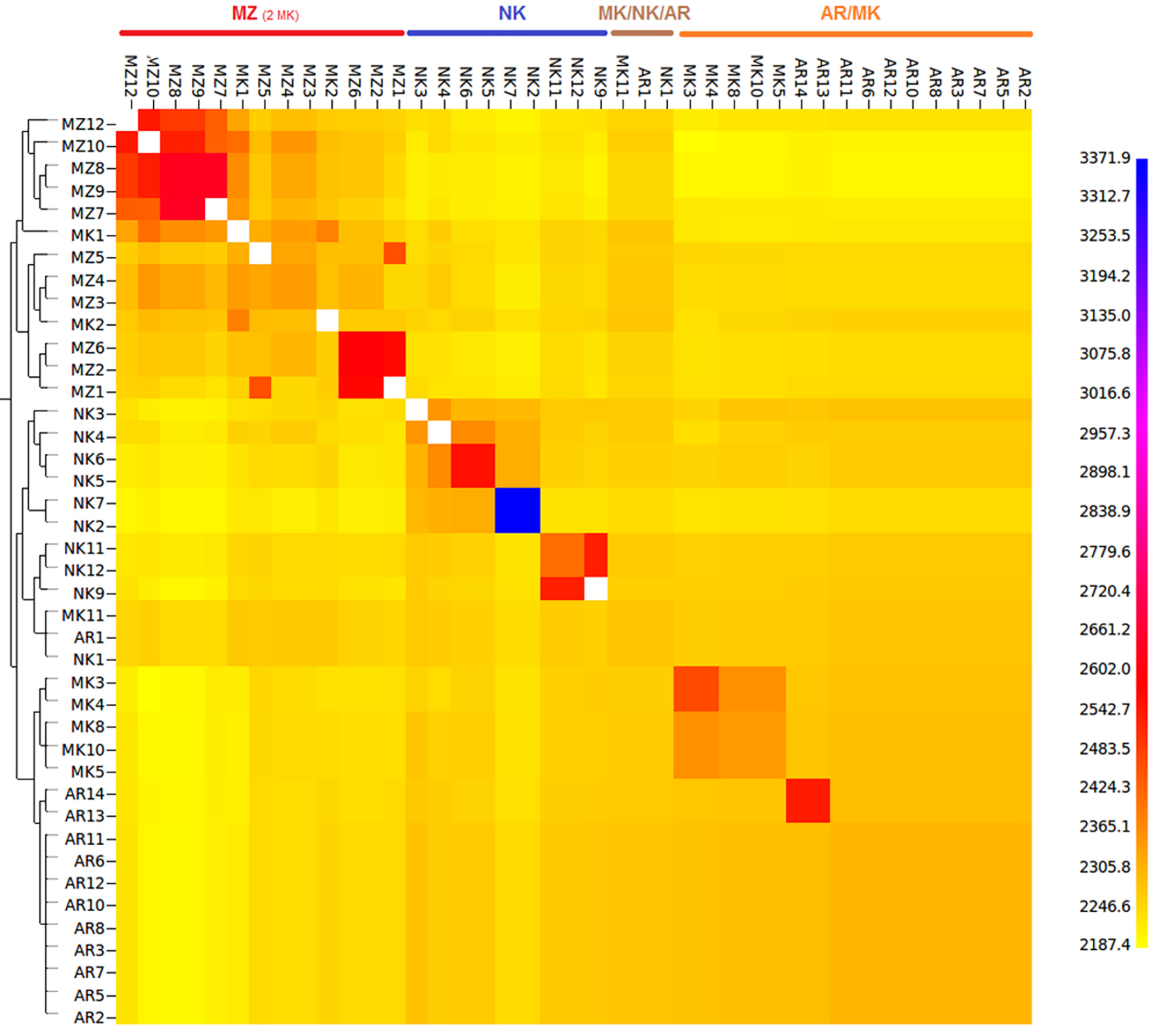
FineSTRUCTURE clustering for four Algerian goat breeds. The color of each bin in the matrix indicates the number of “genomic chunks” copied from a donor (column) to a recipient individual (row). The following abbreviations are used in the figure: MZ = M’Zabite; NK = Kabyle; AR = Arabia; MK = Makatia.

The co-ancestry heatmap (Fig 2) obtained with CHROMOPAINTER/fineSTRUCTURE presents the number of shared genomic “chunks” between the different Algerian individuals. The darker/bluish colors indicate higher co-ancestry estimates, the yellower colors the lower ones. M’Zabite appeared separated from other populations by the number of shared haplotypes. It should be however noted that two Makatia clustered with M’Zabite which is consistent with their genetic proximity observed in Fig 1A and 1B. The heatmap showed that Arabia and Makatia were closely related. Kabyle was characterized by the greatest heterogeneity with, on one side, two individuals (NK7 and NK2, *i*.*e*. same individuals as those distinguished in Fig 1A and 1B) showing the highest number of genomic chunks shared of the dataset (in dark blue), whereas, on the other side, some individuals (*e*.*g*. NK1) clustered with Arabia and Makatia.

**Fig 3:**
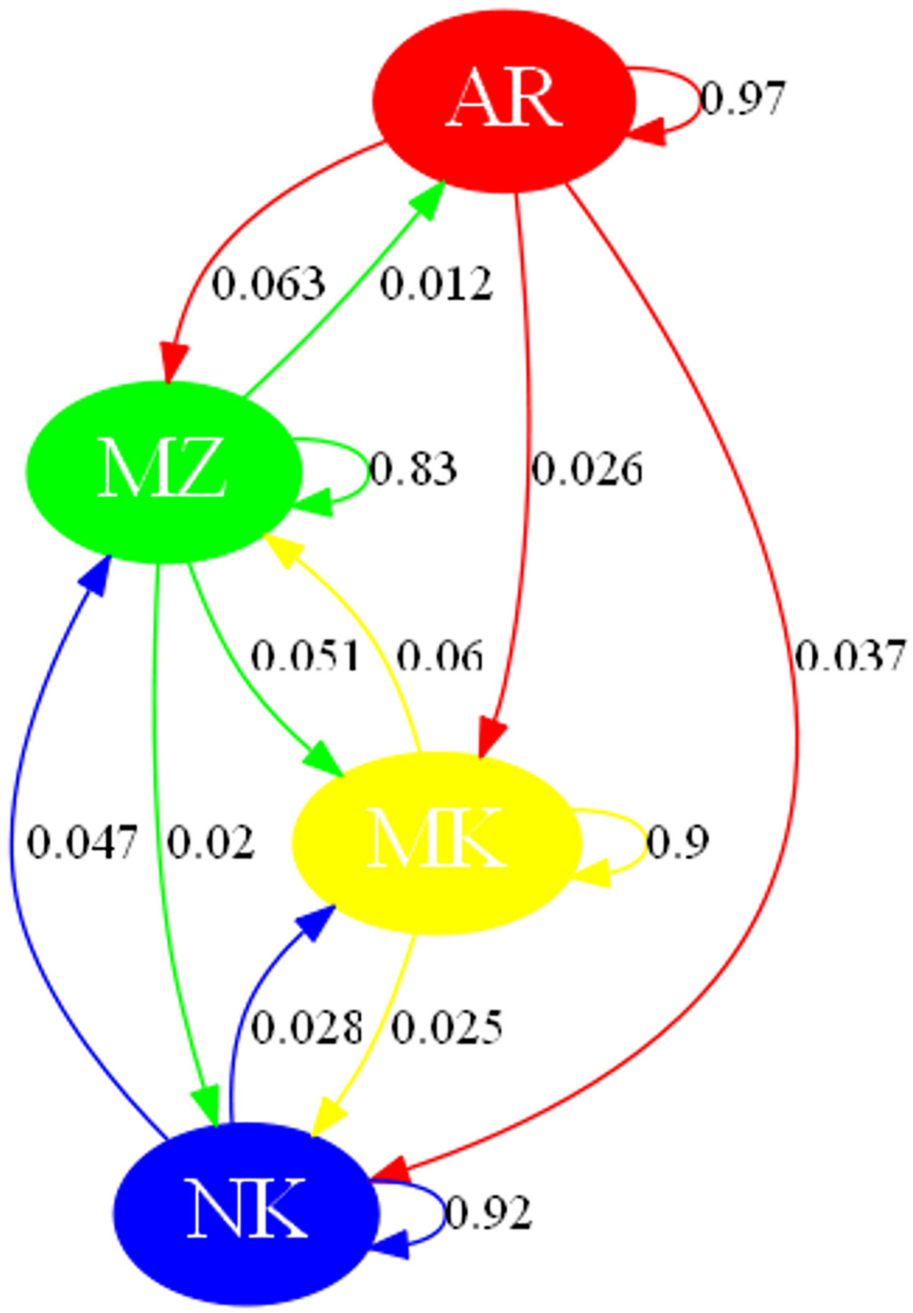
Gene flow network obtained from BAPS analysis and considering goat breeds of Algeria. Self-looping arrows represent the own genetic sources of the population. Gene flows inferior to 0.01 were not displayed due to pruning in order to improve readability of the figure. The following abbreviations are used in the figure: MZ = M’Zabite; NK = Kabyle; AR = Arabia; MK = Makatia.

The BAPS analysis of Algerian breeds, allowed visualization of gene flow (Fig 3). It highlighted the dominant position of Arabia in the Algerian network with a value of self-looping close to 1, indicating that its gene pool was only scarcely influenced by other breeds. Furthermore, gene flows radiating from this cluster toward Makatia, M’Zabite and Kabyle underlined the large Arabian influence on the overall Algerian make-up.

#### Comparison of Algerian and Moroccan goats

**Fig 4:**
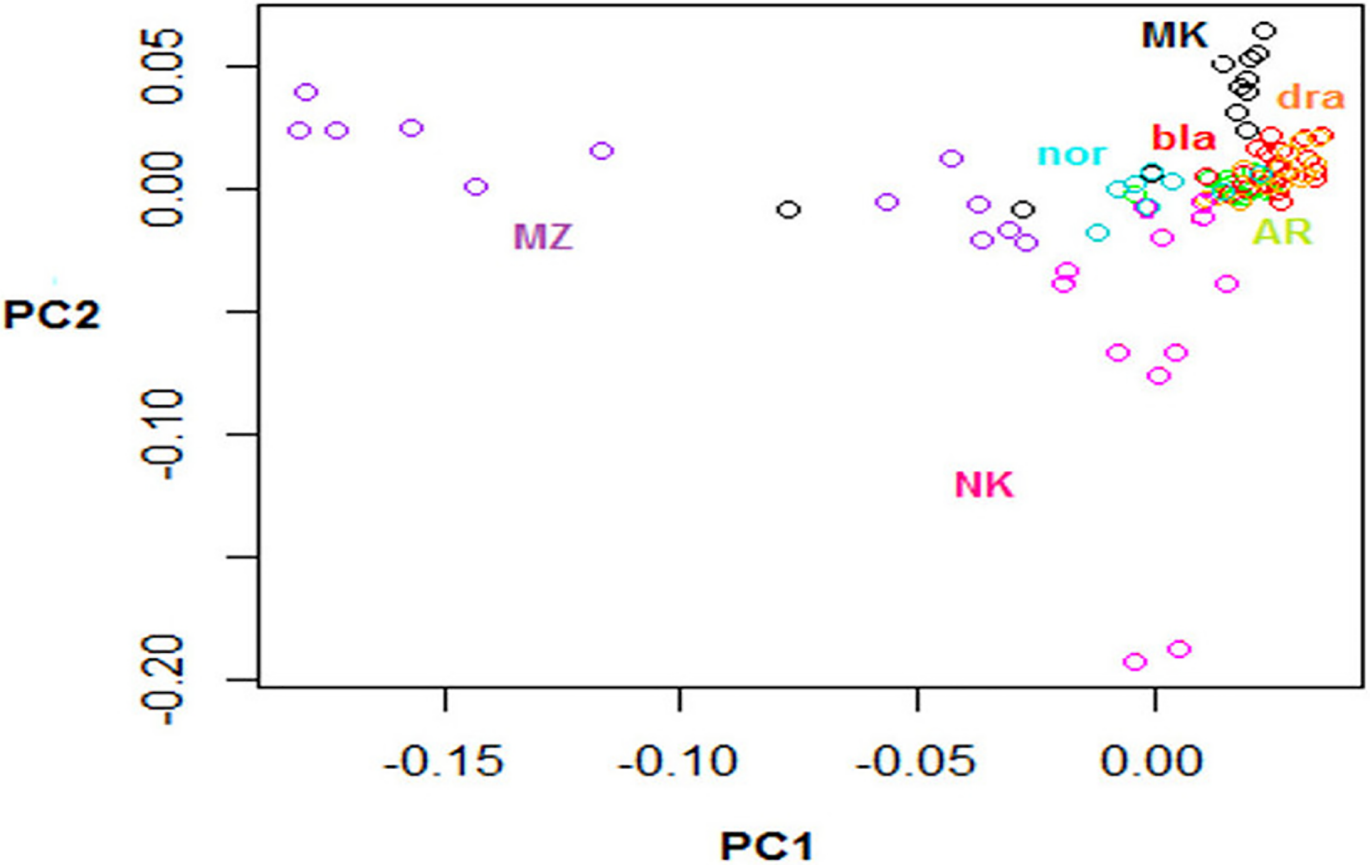
Multidimensional scaling analysis based on the matrix of pair-wise Identity By State (IBS) similarity scores considering both Algerian and Moroccan breeds. The following abbreviations are used in the figure: MZ = M’Zabite; NK = Kabyle; AR = Arabia; MK = Makatia, bla = Black, dra = Draa, nor = Nord.

MDS analysis of Algerian and Moroccan goats (Fig 4) showed that Black, Draa and Arabia were indistinct; Nord was really close to this central core; Makatia was slightly more distant with two individuals clustering with M’Zabite. Kabyle and M’Zabite were again clearly differentiated with a few Kabyle close to Arabia, Black and Draa.

**Fig 5:**
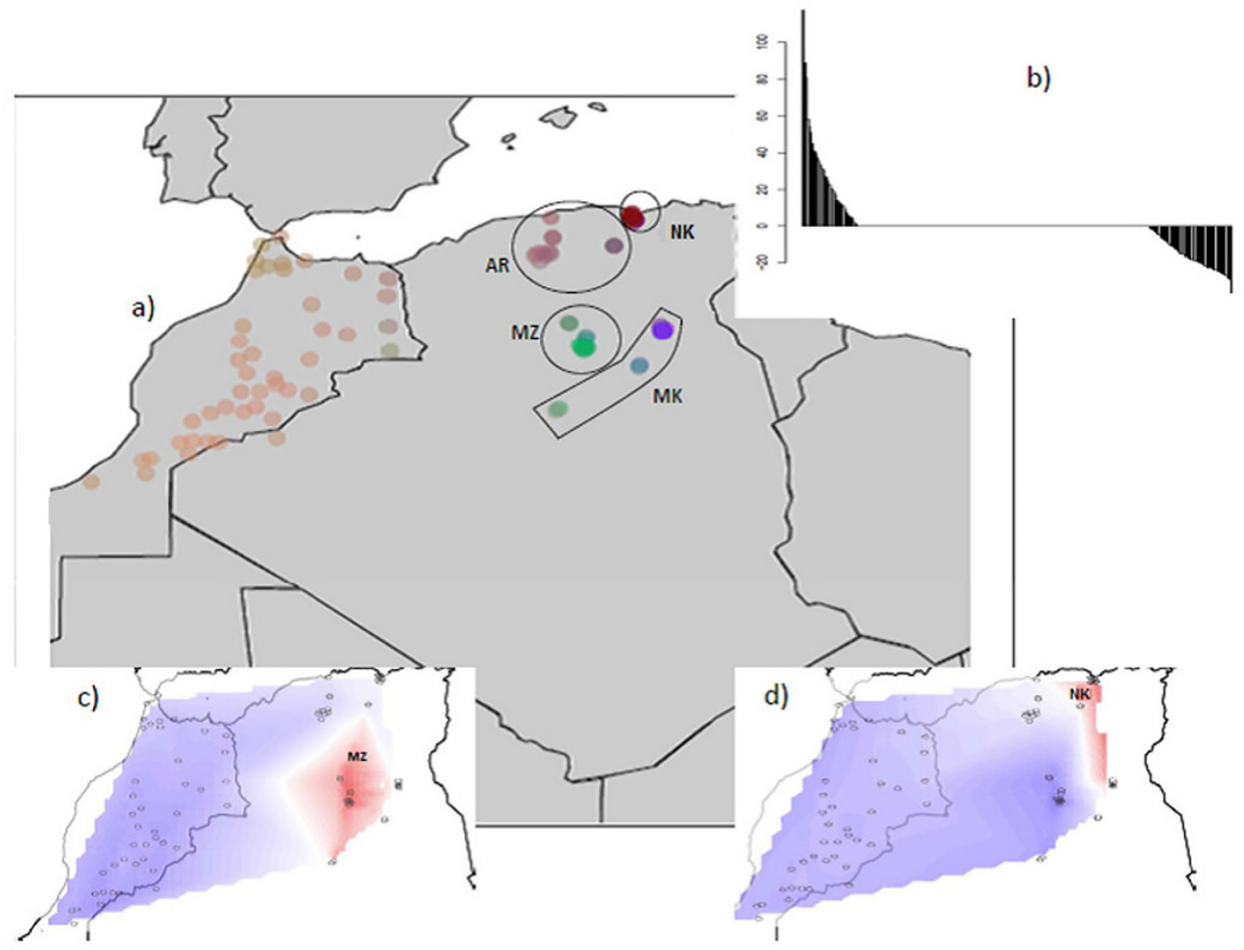
a) Spatial PCA (sPCA) Analysis of Algerian and Moroccan goat breeds; b) eigenvalues for each global and local axis (positive and negative eigenvalues indicate global and local structures, respectively); c) first global score of sPCA; d) second global score of sPCA.

In the sPCA analysis, the consideration of eigenvalues suggested the possibility of a spatial pattern, as the three first positive scores were distinguished from other eigenvalues (Fig 5B). The global Monte Carlo test (10,000 iterations) confirmed significant global spatial structure (p-value = 0.018) and also significant local spatial structure (p-value = 0.012). The Mantel test showed correspondence between geographic and genetic distances (p-value = 0.044). Plotting the first three coordinates confirmed a clear homogeneity of the most Western part of the Maghrebin stock (Fig 5A) and also highlighted the genetic distinctness of M’Zabite and Kabyle, respectively shown by first and second components, (Fig 5C and 5D) and to a lesser extent that of Makatia.

**Fig 6:**
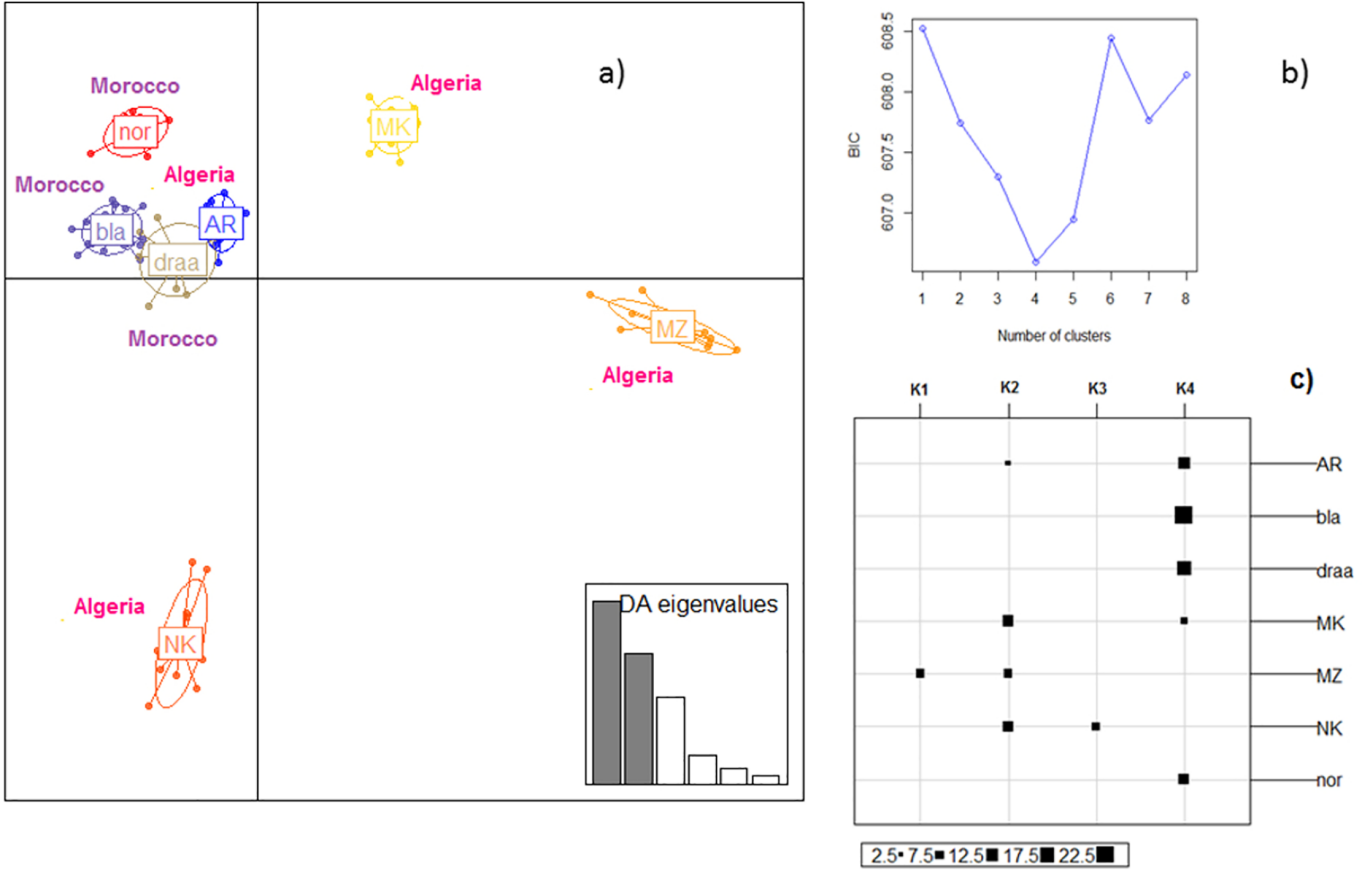
a) Scatterplot of the first two principal components of genetic DAPC using breeds as prior clusters. Breeds are labeled inside their 95% inertia ellipses, and dots represent individuals. The inset indicates the eigenvalues of the first principal components; b) Value of BIC versus number of clusters; c) individual assignment into the four clusters defined without *a priori*. The following abbreviations are used in the figure: MZ = M’Zabite; NK = Kabyle; AR = Arabia; MK = Makatia, bla = Black, dra = Draa, nor = Nord.

In the DAPC analysis, 70 PCs of the PCA were retained as input to DA, accounting for approximately 70% of the total genetic variability. The scatterplot of the first two components of the DA (Fig 6A) confirmed the MDS and sPCA picture: *i*.*e*. the genetic relatedness of Arabia, Draa, Black and Nord and distinct positions of M’Zabite, Kabyle, and to a lesser extent of Makatia. Considering the analysis conducted without prior breed information (Fig 6B), the Bayesian Information Criterion (BIC) indicated 4 clusters: K1 was entirely composed of M’Zabite, and K3 of Kabyle; K4 was a mixture of Arabia, Makatia, Black, Draa and Nord; K2 was a mixture of the remaining individuals, exclusively of Algerian origin (Fig 6C).

## Discussion

Our comprehensive analysis of a genome-wide SNP dataset provided original information on the genetic diversity of Algerian goat breeds, in a national and trans-boundary context, and assessed the extent to which genetic homogenization affected the Maghrebin goat stock.

### Diversity and structure of Algerian goat

The MDS (Fig 1A), STRUCTURE (Fig 1B) and fineSTRUCTURE (Fig 2) analyses showed a good consistency and indicated the following: First, phenomenon of genetic dilution clearly affected Algerian goat stock. Arabia and Makatia, with the lowest pair-wise F_ST_ value, appeared to be the most admixed. These breeds, belonging to Arab type (some authors supposed that Makatia derived from Arabia [17]), show overlapping of their distribution areas, which could favor crossbreeding. In addition, the patterns suggested genetic exchanges between Arabia and Kabyle and to a lesser extent, between Arabia and M’Zabite. Second, in spite of this general trend, M’Zabite and Kabyle preserved clear genetic distinctness. The Kabyle breed was particularly characterized by inter-individual variability, with some individuals strongly affected by admixture.

Monitoring of Algerian goat breeds is almost nonexistent. The most recent census date back to more than ten years, and in particular, the number of Makatia heads is “unknown” (S1 Table). This study indicates that cross-breeding is common mainly with Arabian goats, which are the most numerous. Indeed, this hardy goat is appreciated by breeders because of its adaptation to steppe areas and its large body conformation [45]. The current state of Algerian goat stock is reminiscent of that of Algerian sheep, for which strong gene flows between breeds have also been indicated [21–22]. However, while the situation for the Algerian sheep stock appeared critical with the ancestral Berber sheep breed being absorbed by the Ouled-Djellal breed, the situation of the Algerian goat stock does not seem to be as alarming. All pairwise F_ST_ values of the goat dataset were significantly different from zero (Table 1), which was not the case for the Algerian sheep dataset; moreover, MDS and sPCA showed only limited overlap of the Algerian goat breeds.

### Algerian goat breeds in a trans-boundary context

The average observed heterozygosity (H_o_) of Algerian goats was of 0.40. Comparisons with other studies, using the GoatSNP50K BeadChip, lead to conclude that high level of genetic diversity existed in Algeria. Indeed, study of Nicholoso *et al*. [46] considering 14 Italian goat breeds showed values of H_o_ ranging from 0.35 to 0.41; Manunza *et al*. [47] recorded values of H_o_ ranging from 0.35 to 0.42 considering seven Spanish goat breeds (with an exception for Palmera, displaying a value of 0.28); the study of Kim *et al*. [48] found a value of H_o_ of 0.40 for the Barki Egyptian breed.

Considering both Algeria and Morocco, MDS (Fig 4) and sPCA (Fig 5) showed that the genetic peculiarity of M’Zabite and Kabyle was still obvious on this larger scale, whereas homogeneity between the remaining Moroccan and Algerian breeds was evident. In particular, the Algerian Arabia and the Moroccan Black, Draa and Nord appeared as a single and homogeneous genetic group. This was also supported by a mean F_ST_ value close to zero, and by sequential K-means clustering results (Fig 6C).

M’Zabite and Kabyle were the most distinct breeds in our study. M’Zabite is an old breed, linked to the Nubian type, which evolved under singular conditions. Indeed, it was reared by the Mozabites, a Berber ethnic group, which took refuge in the Mzab region, in the 13^th^ century. Interestingly, studies on human genetics revealed a genetic uniqueness of the Mozabites, shaped by various migrations from neighboring regions [49]. Gene flows from Near East, Arabic migrations across North Africa 1,400 years ago, and also trans-Saharan transports of slaves from sub-Saharan Africa were identified as key migratory movements underlying the genetic pattern of Mozabites [49].

The Kabyle breed, belonging to Berber type, was probably the first wave of goat migration from Near-East around 6000 BP. It was kept by the Kabyle Berber people, who were largely independent during the Ottoman Empire rule and have thereafter shown strong resistance to French colonization. Like the Mozabites, they lived in relative isolation over centuries, given the strong political tensions that still persist today.

The weak differentiation between the Moroccan breeds was explained by Benjelloun *et al*. [15] by numerous waves of migrations along different routes (*e*.*g*. Mediterranean, North-African [6], [50]), coupled with high levels of gene flow, soft selection pressure, and the absence of strong population bottlenecks during the breed formation. All these elements would have led to the observed pattern, characterized by homogeneous populations with high genetic diversity. An alternative hypothesis could be stated, considering more particularly the Berber breeds case. Indeed, the Berber type spread over Moroccan High-Atlas around 6000 BP [10]. This may have led to the emergence of the Black breed, also called “Black of Atlas”, classified in the Berber type [51]. Reports of the “Livestock Production Service” by French colonists, mainly agronomists, described, in the early 20th century [52–55], the Moroccan Berber breeds (sheep and goat) as well-defined and phenotypically characterized, suggesting clear genetic identity for these breeds. The same applies to the Algerian Berber breeds, which were described in details during same periods [16], [56]. In view of these elements, homogeneity between Moroccan breeds and in particular between Black (Berber type from Morocco) and Arabia (Sahelian type from Algeria) may result from cross-breeding which occurred after breed formation and mainly during the last centuries. In Algeria, Couput [57] already reported in 1900, the growing absorption of Berber breeds by Arabian breeds, while only isolated Berber populations (*e*.*g*. in mountains) remained “pure”. Under this scenario, Algeria would represent an early stage of the homogenization process, with breeds, from Arab or Berber origin, still distinct from a genetic point of view, while Morocco may have reached final stages. Further studies, including historical archives and archeogenetic researches, are needed to really understand the trajectory followed by Maghrebin breeds and depict which scenario is more likely. In addition, it would be relevant to clarify the impact of “exotic” breeds on the indigenous stock, and also in the homogenization process. Indeed, although “exotic” breeds are today mainly represented by Saanen and Alpine in the Maghreb area, during the last centuries, imports of different breeds from adjacent Mediterranean regions (Spain, Malta, *etc*.) have been widely reported. In Algeria for example, Maltese goat presence was already reported in 1857 [58]. In Morocco, the Spanish protectorate (1912–1956) favored introduction of Spanish breeds (Murciana Granadina, Malaguiña, *etc*.), and their crossing with indigenous breeds, particularly in the north of the country [19].

In conclusion, this study highlighted the clear genetic dilution affecting the Algerian stock and a global homogeneity considering both Algeria and Morocco, *i*.*e*. a part of the North African reservoir of diversity shaped by long and complex history. The “livestock revolution” [59] has led to the use of a limited number of breeds for intensive production systems. In developing countries, different strategies have been adopted to increase productivity; in most cases, improved exotic breeds were directly used or crosses were practiced with a limited number of more productive breeds (exotic or local). In this study, we pointed out the possible role of crossbreeding practices in the loss of genetic integrity of Maghrebin breeds. Such results imply the implementation of measures to improve our still limited understanding of the North-African goat stock, in order to preserve this early livestock of Africa.

## Supporting information

S1 Table Breed details for the four Algerian goat breeds.
(DOCX)

S2 Table Genetic diversity, considering four Algerian goat breeds.
(DOCX)
