# Supplementary material for: Genetic homogeneity of North-African goats
Source: PLoS One. 2018 Aug 16;13(8):e0202196. doi: 10.1371/journal.pone.0202196 (PMC6095539; doi:10.1371/journal.pone.0202196)
Supplement: S1 Table — Breed details for the four Algerian goat breeds. (DOCX) [file pone.0202196.s001.docx]

| **Breed and Type (according to Mason^1^)** | **Geographic location** | **Population in number of heads (date of last record)** | **Description/traits** | **Region** | **Sampling localization/GPS coordinates (gender and age)** | **Number of breeding establishment** | **Gender**  **(M: male/**  **F: Female)** | **Age**  **(in years)** |
| --- | --- | --- | --- | --- | --- | --- | --- | --- |
| **Arabia**  (12 individuals)  *Sahelian type* | Steppic areas | 810.000 (1997)  MAP^2^ | -High on leg, large conformation  - Very hardy can remain two days without drinking  -Highly susceptible to trypanosomiasis | Tipaza | 36°34'30.48"N/2°40'52.70"E | 1 | F | 2 |
|  |  |  |  | Tiaret | 35°13'8.49"N/2°19'12.58"E | 2 | F | 3 |
|  |  |  |  |  | 35°23'45.00"N/2°13'55.55"E | 3 | F | 7 |
|  |  |  |  |  | 35°18'11.14"N/2°15'58.63"E | 4 | F | 6 |
|  |  |  |  | Djelfa | 35°19'7.02"N/2°33'34.66"E | 5 | F | 4 |
|  |  |  |  |  | 35°25'55.70"N/2°39'7.21"E | 6 | F | 6 |
|  |  |  |  |  | 35°24'34.11"N/2°22'51.50"E | 7 | M | 4 |
|  |  |  |  |  | 35°25'55.70"N/2°39'7.21"E | 8 | F | 4 |
|  |  |  |  | Médéa | 35°53'18.62"N 2°44'59.69"E | 9 | M | 2 |
|  |  |  |  |  | 35°52'29.24"N 2°45'4.11"E | 10 | F | 2 |
|  |  |  |  | Msila | 5°36'51.89"N 4°31'18.11"E | 11 | M | 1.5 |
|  |  |  |  |  | 35°42'53.47"N 4°32'10.42"E | 12 | M | 1.5 |
| **Makatia**  (12 individuals)  *Sahelian type* | Highlands and Northern Algeria | unknown | - Very hardy can remain two days without drinking  - Highly susceptible to trypanosomiasis | Ghardaia | 32°30'18.41"N/3°37'38.73"E | 1 | F | 1 |
|  |  |  |  |  |  |  | M | 1 |
|  |  |  |  |  |  |  | F | 5 |
|  |  |  |  |  | 32°28'22.91"N/3°41'43.01" | 2 | F | 6 |
|  |  |  |  |  |  |  | F | 6 |
|  |  |  |  |  | 32°49'20.51"N/3°46'6.68"E | 3 | F | 6 |
|  |  |  |  |  |  |  | F | 5 |
|  |  |  |  |  | 32°28'20.80"N/3°44'47.18"E | 4 | F | 1 |
|  |  |  |  |  |  |  | F | 5 |
|  |  |  |  |  |  | 5 | F | 5 |
|  |  |  |  |  | 33°16'15.60"N/3°11'16.97"E | 6 | F | 5 |
|  |  |  |  |  |  |  | F | 5 |
| **M’zabite**  **(« Oasis red goat » or « Touggourt »)**  (12 individuals)  *Nubian type* | Originated from M’tlili can now be found throughout  the northern part of Sahara | 704.000 (2004)  FAO DAD-IS^3^ | - Able to handle stressful conditions characteristic of oasis (Sahelian conditions)  - Prolific | Ghardaia | 30°35'35.63"N/2°52'38.00"E | 1 | F | 3 |
|  |  |  |  |  |  | 2 | F | 2 |
|  |  |  |  | Ouargla | 33° 6'40.19"N/ 6° 4'20.07"E | 3 | F | 4 |
|  |  |  |  |  |  | 4 | F | 5 |
|  |  |  |  |  | 33° 0'42.13"N/6° 0'36.12"E | 5 | F | 1 |
|  |  |  |  |  |  |  | F | 6 |
|  |  |  |  |  |  | 6 | M | 2 |
|  |  |  |  |  | 33° 0'52.44"N/ 6° 1'16.36"E | 7 | F | 4 |
|  |  |  |  |  |  | 8 | F | 4 |
|  |  |  |  |  |  |  | F | 1 |
|  |  |  |  |  | 31°57'46.68"N/5°20'9.22"E | 9 | F | 2 |
|  |  |  |  |  |  | 10 | F | 1 |
| **Kabyle**  **(« Naine de Kabylie »)**  (12 individuals)  *Berber type* | Mountainous  regions of Kabylia and Aures Dahra | 427.500 (1992)  FAO DAD-IS^3^ | - Reduced conformation (dwarf)  - Well suited to the mountainous regions of the northern Atlas Mountains | Bejaia | 36°28'16.24"N/5°13'11.80"E | 1 | F | 3 |
|  |  |  |  |  |  |  | F | 3 |
|  |  |  |  |  | 36°25'3.94"N/5°13'27.49"E | 2 | F | 2 |
|  |  |  |  |  |  |  | F | 3 |
|  |  |  |  |  | 36°36'26.92"N/5°11'1.11"E | 3 | F | 2 |
|  |  |  |  |  |  | 4 | F | 4 |
|  |  |  |  | Setif | 36°30'50.63"N/5° 7'6.68"E | 5 | M | 1 |
|  |  |  |  |  |  | 6 | F | 1 |
|  |  |  |  |  | 36°29'51.44"N/5° 6'10.87"E | 7 | F | 2 |
|  |  |  |  |  |  |  | M | 1 |
|  |  |  |  |  |  | 8 | M | 1 |
|  |  |  |  |  |  |  | F | 1 |

1:Mason IL (1969). A Dictionary Of Livestock Breeds

2:MAP, Ministère de l’Agriculture et de la pêche, (1998). Statistiques des productions animales de l’année 1997 MAP, Alger.

3:FAO DAD-IS database: www.fao.org/dad-is
