# Supplementary material for: Genetic homogeneity of North-African goats
Source: PLoS One. 2018 Aug 16;13(8):e0202196. doi: 10.1371/journal.pone.0202196 (PMC6095539; doi:10.1371/journal.pone.0202196)
Supplement: S2 Table — Genetic diversity, considering four Algerian goat breeds. (DOCX) [file pone.0202196.s002.docx]

| Breed | n | P | Nb. of SNP not conform to quality filters* | H_o_ (s.d.) |
| --- | --- | --- | --- | --- |
| Makatia | 12 | 0.98 | 5982 | 0.37 (0.17) |
| Arabia | 12 | 0.98 | 4483 | 0.41 (0.17) |
| M’Zabite | 12 | 0.99 | 5751 | 0.42 (0.16) |
| Kabyle | 12 | 0.98 | 5758 | 0.41 (0.18) |

**n**. sample size; **P**. proportion of polymorphic loci; **Nb**. number; **H_o_**. observed heterozygosity; *see Material and Methods.
